# A comparative study of the biosynthesis of CuNPs by *Niallia circulans* G9 and *Paenibacillus* sp. S4c strains: characterization and application as antimicrobial agents

**DOI:** 10.1186/s12934-024-02422-0

**Published:** 2024-05-28

**Authors:** Nahla M. Abdel Aziz, Doaa A. Goda, Dina I. Abdel-Meguid, Ebaa E. EL-Sharouny, Nadia A. Soliman

**Affiliations:** 1 Botany and Microbiology Department, Faculty of Science, Alexandria University, 21526, Alexandria, Egypt; 2 Bioprocess Development Department, Genetic Engineering and Biotechnology Research Institute (GEBRI), City of Scientific Research and Technological Applications (SRTA-City), Universities and Research Institutes Zone, P.O. 21934, New Borg El-Arab City, Alexandria, Egypt

**Keywords:** Biogenetic, Copper, Nano particles, Characterization, Biological activity, Candida

## Abstract

**Background:**

Biosynthesis of metallic nanoparticles using microorganisms are a fabulous and emerging eco-friendly science with well-defined sizes, shapes and controlled monodispersity. Copper nanoparticles, among other metal particles, have sparked increased attention due to their applications in electronics, optics, catalysis, and antimicrobial agents.

**Results:**

This investigation explains the biosynthesis and characterization of copper nanoparticles from soil strains, *Niallia circulans* G9 and *Paenibacillus* sp. S4c by an eco-friendly method. The maximum reduction of copper ions and maximum synthesis CuNPs was provided by these strains. Biogenic formation of CuNPs have been characterized by UV–visible absorption spectroscopy, X-ray diffraction, Fourier transform infrared spectroscopy, X-ray analysis and transmission electron microscopy analysis. Using UV-visible spectrum scanning, the synthesised CuNPs’ SPR spectra showed maximum absorption peaks at λ_304&308 nm_. TEM investigation of the produced CuNPs revealed the development of spherical/hexagonal nanoparticles with a size range of 13–100 nm by the G9 strain and spherical nanoparticles with a size range of 5–40 nm by the S4c strain. Functional groups and chemical composition of CuONPs were also confirmed. The antimicrobial activity of the biosynthesized CuNPs were investigated against some human pathogens. CuNPs produced from the G9 strain had the highest activity against *Candida albicans* ATCC 10,231 and the lowest against *Pseudomonas aeruginosa* ATCC 9027. CuNPs from the S4c strain demonstrated the highest activity against *Escherichia coli* ATCC 10,231 and the lowest activity against *Klebsiella pneumonia* ATCC 13,883.

**Conclusion:**

The present work focused on increasing the CuNPs production by two isolates, *Niallia circulans* G9 and *Paenibacillus* sp. S4c, which were then characterized alongside. The used analytics and chemical composition techniques validated the existence of CuONPs in the G9 and S4c biosynthesized nano cupper. CuNPs of S4c are smaller and have a more varied shape than those of G9 strain, according to TEM images. In terms of antibacterial activity, the biosynthesized CuNPs from G9 and S4c were found to be more effective against *Candida albicans* ATCC 10,231 and *E. coli* ATCC 10,231, respectively.

## Background

Now-a-days, biological methods or green methods are gaining impetus as chemical or physical methods are not economic and safe for ‘in vivo’ use [1], also these methods involve a large amount of heat and energy, use of elevated temperature and toxic chemicals [2]. Thus, development of clean, non-toxic, environment-friendly and biocompatible methods for the synthesis of nanoparticles is needed, many microorganisms (bacteria, fungi, actinomycetes, algae and viruses) have been investigated to produce different metal nanoparticles of silver, gold, zinc, copper, titanium, alginate, and magnesium [3, 4]. The use of biological systems as a green synthesis approach results in the development of stable, distributed, and size-controlled nanoparticles with desirable physicochemical properties [5, 6] and exquisite morphology at ambient conditions [7]. Biosynthesis of metal nanoparticles in living system is catalyzed by various reducing agents and/or reductase-type enzyme present intracellularly or extracellularly [8].

Among all biological systems used till now, bacteria have acquired significant attention [9] as they are easy to culture, produce extracellular nanoparticle. Also, it requires mild experimental conditions like pH, temperature, have easy downstream processing and short generation time for nanoparticle synthesis [10]. In a study by He et al. [11], it was discovered that changing the pH of the growth medium during incubation resulted in production of nanoparticles of differing size and shape. Controlling such properties is important, as varying sizes of nanoparticles are required for different applications such as optics, catalysts or anti-microbials.

In nature, bacteria are frequently exposed to diverse and sometimes extreme environmental situations. Survival in these harsh conditions ultimately depends on their ability to resist the effects of environmental stresses. Natural defence mechanisms exist in bacteria to deal with a variety of stresses such as toxicity arising from high concentrations of metallic ions in the environment, as they able to change the metal ion concentration via redox state changes, efflux systems, intracellular precipitation, and accumulation of metals, and extracellular formation of complexes [12].

Cu NPs have attracted a lot of attention among group IB metals because of its unique properties and potential applications as electronic materials, catalysts, lubricants, thermal transfer nanofluids, nanocomposite coatings, and optical devices [13], in addition to its significantly lower cost when compared to silver and gold nanoparticles. Numerous techniques, such as chemical and physical procedures, could be utilized to synthesize CuNPs [14, 15]. However, the majority of these techniques require sophisticated instruments, biologically hazardous substances, and an oxygen-free atmosphere in order to be successful. Reports on the synthesis of CuNPs without an inert environment are extremely rare [16]. Therefore, more robust, economical, and environmentally friendly methods are still needed to synthesis stable CuNPs.

Usha et al. [17] reported a green synthesis of copper oxide by *Streptomyces* sp for development of antimicrobial textiles which can be useful in hospitals to prevent or to minimize infection with pathogenic bacteria. Singh et al. [18], reported biological synthesis of copper oxide nanoparticles using *Escherichia coli* (*E. coli*) with a variable size and shapes. *Serratia* sp. ZTB29 [19], *Morgenella* species [20], *Stenotrophomonas* sp. BS95 [21] are also some examples for the bacteria that are able to synthesis CuNPs.

Copper nanoparticles (CuNPs) are considered as candidates for antimicrobial applications as biocides, and antibiotic treatment; these have been shown to hinder the growth of bacteria like *E. coli, Bacillus subtilis* and plant pathogen [22, 23]. Also it can stimulate the plant growth [19] and degrade the pesticide residues [23].

The release of Cu^2+^ ions is the widely approved process by which copper-based nanomaterials exhibit antibacterial activity. Copper ions have the ability to harm bacterial cell membranes, penetrate cells and impair the enzyme machinery [24]. Because of their high surface-to-volume ratio, copper nanoparticles are extremely reactive antimicrobial materials. Since CuO NPs are the most basic member of the copper group, they are among the most significant metal oxide nanoparticles. CuO NPs have significant antibacterial characteristics due to their structure, which prevent the growth of bacteria, fungus, viruses, and algae [25]. Highly effective biocidal materials, copper-based compounds are frequently utilized in pesticide formulations and other health-related applications [26]. In addition the biosynthesized NPs can be stabilized by coating of low-molecular weight biomolecules around the nanoparticles [27]. Yoosefi Booshehri et al. [24] provide a simple process for depositing copper oxide nanoparticles on cellulose paper to form a stable layer of CuO that may be performed in *situ*. Using this approach, copper oxide can be applied to any wettable substrate that can survive a relatively high pH of 10–12 for several hours. CuO was placed on commercial fabrics and polyethersulfone flat sheets using the same method. The resulting CuO-paper is more effective against both + Ve and –Ve microorganisms. The release of copper ions from CuO nanoparticles on the paper provides the antibacterial effect. The finished composite can be used as an affordable point-of-use system to produce clean, safe drinking water.

**Fig. 1:**
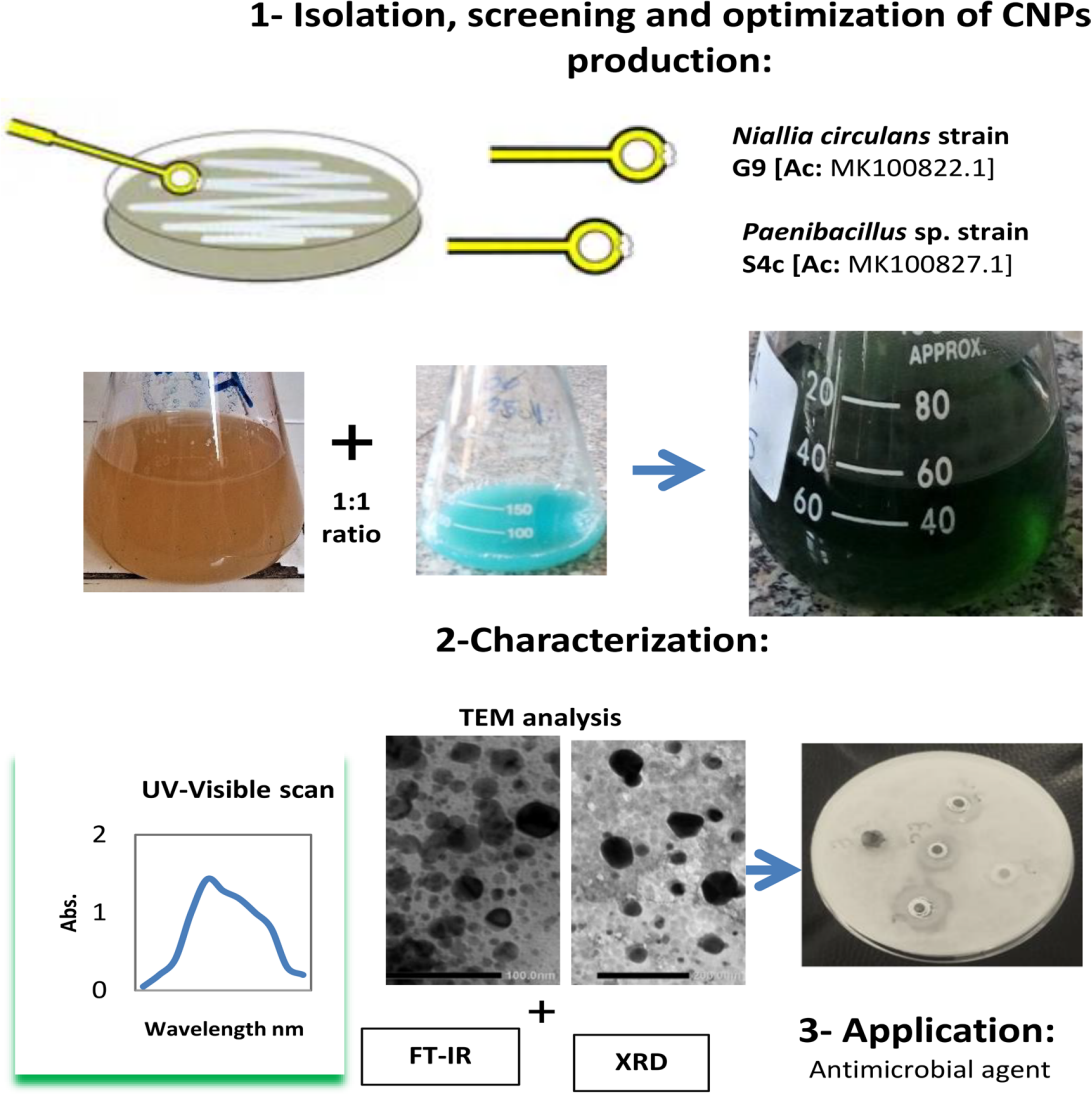
Schematic outline of CuNPs biosynthesis

Based on the prospective properties and uses of CuNps, the purpose of the current study was to screen various Egyptian bacterial isolates as model biological systems to synthesize and analyze CuNPs. Furthermore, the biosynthesized Cu-NPs’ potential use as antibacterial agents was explored. This is the first study to disclose the green synthesis of CuNPs by *Niallia circulans* G9 and *Paenibacillus* sp. S4c strains, and the overall schematic design for the work is given in Fig. 1.

## Materials and methods

### Isolation, culturing, and screening for CuNPs synthesis

All microbes used in this work were isolated from an Egyptian agricultural soil. All strains were grown on liquid Luria–Bertani (LB) medium (g/L): Tryptone 10, yeast extract (YE) 5, and NaCl 5, at temperature (T) 37 °C and pH 7.0 under shaking (200 rpm) for 48 h. The culture supernatants from these strains were obtained by centrifugation (10,000 rpm, 4 °C, 10 min) under sterile condition and incubated with 50 mM CuSO4 at 1:1 volume ratio. The biosynthesis of CuNPs was observed visually by the color change from blue to dark green after incubation for 24–48 h at 37 °C and 120 rpm.

### Spectrophotometric detection for CuNPs

Preliminary formation of CuNPs was detected by monitoring absorption spectra in the wavelength range of 200–800 nm using of UV-Visible spectrophotometer [28]. A non-inoculated LB medium with 100 mM CuSO4 was maintained as control.

### Nitrate reductase (NR) activity assay

NR activity was measured according to Vaidyanathan et al. [29]. The absorbance of pink color appeared was recorded at 540 nm. Enzyme units are defined as micromoles of nitrite produced per min. Nitrite standard curve was prepared by using different concentrations of sodium nitrite (1–10 µM), where 1 µM of nitrite equivalent to 1U.

NR activity was detected in the supernatants of the selected isolates upon culturing in NR- enhanced medium. This medium [30] contained (g/L): Glucose, 10; sucrose, 1; YE, 10; tryptone, 1; ammonium molybdate, 1; NaNO_3_, 1; NH_4_Cl, 0.1; CuSO_4_, 0.01; FeCL_3_,0.01; NaCl, 1; pH 7 and incubated at 37 °C for 48 h under shaking (200 rpm). Afterwards, the culture supernatant was incubated with CuSO4 as mentioned previsously to snthesize the CuNPs, then measured at the recorded λ_uv max_. The measured NR in this medium was compared to LB as well.

### Identification and phylogeny

The finally selected isolates coded G9& S4c were examined morphologically and subjected to molecular identification by amplifying 16S*rRNA* using the universal primers of this gene according to Eden et al. [31]. The sequences of the purified PCR products were determined using the automated fluorescent DNA sequencer [32]. The obtained nucleotide sequence was analysed using BLAST https://blast.ncbi.nlm.nih.gov/ and the phylogenetic analysis was carried out using MEGA X software to compare between the selected strains and the closely related ones.

### Factors impacting the production of CuNP

Biosynthesis of CuNPs depends on various factors like pH, temperature, time of the reaction and concentration of the copper ion. Cell-free supernatant of the culture was exposed to different pHs (3.0, 5.0, 7.0 and 9.0), temperatures (25, 37, 45 and 55 °C), times (12, 24, 48 and 72 h) in presence of copper sulphate, at final concentration 100 mM, to select the optimum conditions for the synthesis of CuNPs. Subsequently, different concentrations of copper sulphate (25, 50, 100 and 150 mM) were tested at the previously preferred conditions for each strain. In all experiments change in the color of the reaction mixtures and surface plasmon resonance (SPR) of synthesizing nanoparticles were monitored to select the most appropriate condition(s).

### CuNPs production

After optimizing the conditions required for CuNPs synthesis by G9 and S4c strains, the maximum yields were obtained by incubating equal volumes of cell free supernatants (overnight culture in NR-enhanced medium) with CuSO4 (100 mM) under these conditions (pH 7.0, 37 °C, 120 rpm & 24 h) and (pH 7.0, 45 °C, 120 rpm & 48 h) for G9 and S4cm, respectively. The colour change in the solution at the end of the incubation period was used to assess the reduction of copper ions.

### CuNPs separation

To recover the formed CuNPs present in the cell-free extracts after incubating with CuSO4, the solution was centrifuged at 15,000 rpm for 20 min. The pellets contain CuNPs were collected with care, re-suspended in double-distilled water (ddH2O), washed severally by repeated centrifugation, freeze-dried (lyophilisation), and then were used for further experimentation.

### CuNPs characterization

#### UV-Visible double beam spectrophotometer

The optical properties of the studied CuNPs were characterized by scanning UV absorption spectra (200–800 nm) using double beam spectrophotometer (Thermo Scientific™ Evolution™ 350 UV-Vis spectrophotometer).

### FT-IR spectroscopy

The FT-IR spectrum of CuNPs samples was analysed using FTIR instrument (Bruker Tensor37), at the Central Laboratory, Faculty of Science, Alexandria University to identify the possible interactions between CuNPs and the biomolecule. Analysis was carried out in the range of 500–4000 cm^− 1^ at the resolution of 1 cm^− 1^ [33]. The synthesized CuNPs sample was lyophilized and diluted with potassium bromide (in the ratio of 1:100) to make a pellet and subjected to study the presence of IR bands.

### EDX analysis

The elemental composition of the synthesised CuNPs (EDX-analysis) was carried out according to Jyoti et al. [34] by using Oxford instrument attach to scanning electron microscope at the Electron Microscope Unit, Faculty of Science, Alexandria University. This analysis done by using powder of lyophilized copper nanoparticles.

### XRD analysis

A monochromatic X-ray diffraction (XRD) beam with wave length lambda was used to analyse the crystalline nature of the biosynthesized CuNPs sample [33]. This analysis was done using Shimadzu XRD7000 instrument at the Central Laboratory, Faculty of Science, Alexandria University operating at 30KV current 10 mA with CuKa radiation (λ = 1.54184 A°) ) in the 2θ range from 20 to 80◦ at a scan rate of 0.03◦ S^− 1^. The fine dried lyophilized powdered of CuNPs were reserved and fixed on a quartz glass slide to make a thin film and then scanned.

### TEM analysis

The produced Cu nanoparticles’ size and morphological characteristics were evaluated using a TEM (JEM-1400 Plus, electron microscope). After diluting and sonicating the CuNPs solution, carbon-coated TEM grids were drop-coated with copper nanoparticles to prepare for TEM investigations. After allowing the film on the TEM grids to dry, the excess solution was wiped off with blotting paper.

### Antimicrobial activity of the synthesized CNPs

The synthesized CuNPs were tested for their antimicrobial activity by the agar well diffusion method [35] against different kinds of human pathogens. The tested strains included; *Staphylococcus aureus* ATCC 25,923 as Gram-positive (G + ve) bacteria, *Pseudomonas aeruginosa* ATCC 9027, *Klebsiella pneumonia* ATCC 13,883 & *E. coli* ATCC 10,231 as Gram-negative (G-ve) bacteria while, *Candida albicans* ATCC 10,231 was tested as yeast. The pathogens were inoculated in LB broth and incubated at 37 °C for 24 h, then swabbed uniformly onto sterile Muller-Hinton Agar (MHA) plates using sterile cotton swabs. Agar wells of 5 mm diameter were prepared with the help of a sterilized stainless-steel cork borer. A 50 µl of different concentrations of copper nanoparticles (60, 80 and 100 µg/ml) were loaded in four wells with the help of micropipette under aseptic conditions. The plates were incubated at 37 °C for 24 h and then the zone of inhibition was measured using a centimeter ruler and the value for each organism was recorded and expressed in millimetre (mm).

### Statistical analysis

Triplex reactions were used for all experiments. Using Microsoft Office Excel 2013, the means ± standard deviation was used to express the results.

## Results

**Fig. 2:**
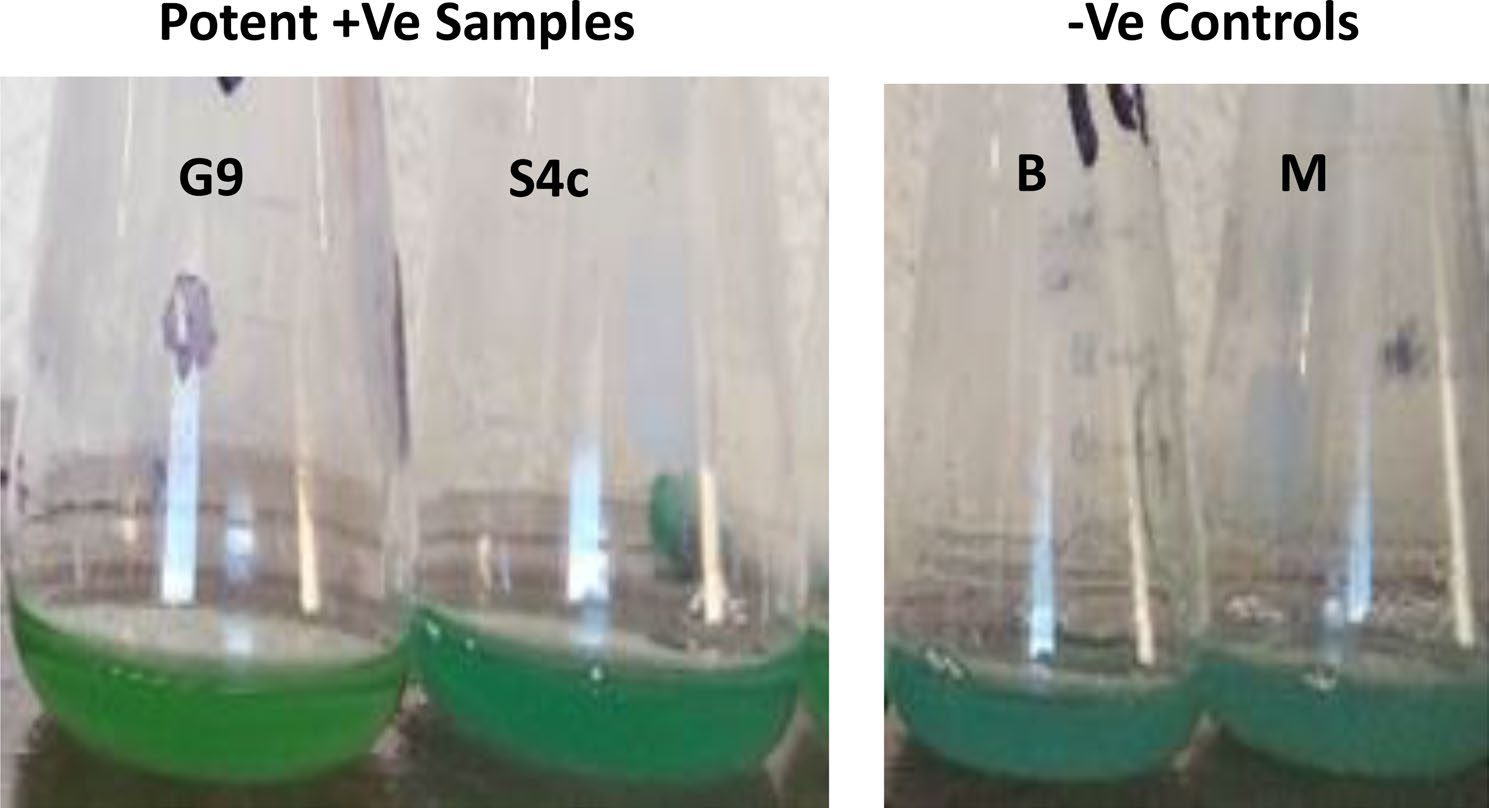
Color change during the reduction of Cupper into CuNPs; the negative controls (B & M) exhibit no change in color, whilst the potent positive samples (G9 & S4c) show a change in color of CuSO4 due to the reduction process into green. *Note: B (-Ve control) comprises 100 mM copper sulphate solution in distilled water, while M (-Ve control) contains non-inoculated medium in copper sulphate (100 mM), with each components mixed in equal proportions

**Fig. 3:**
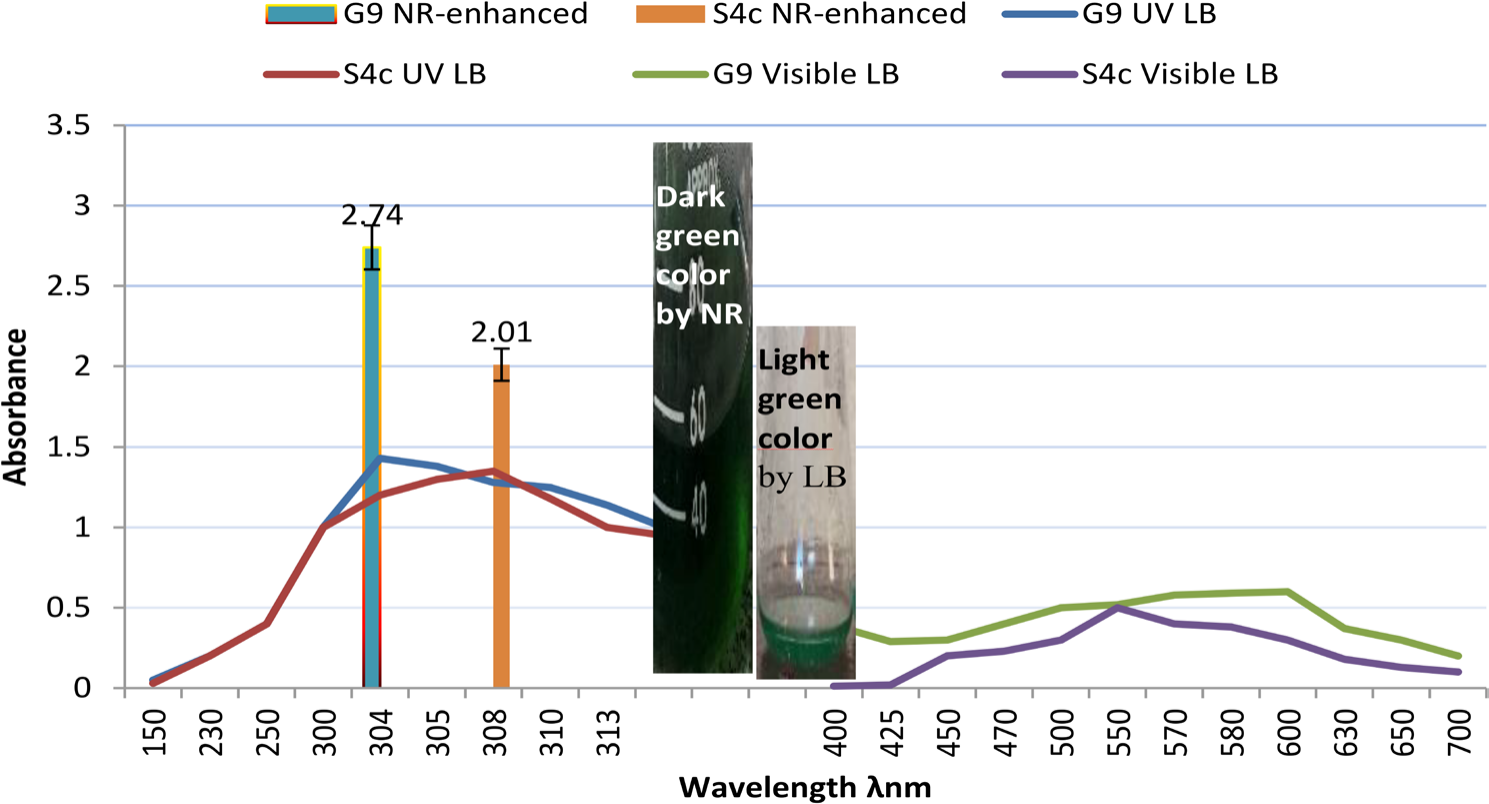
The UV-vis absorption spectra of CuNPs generated by G9 and S4c strains in cell-free supernatant using LB isolation medium, and the recorded absorbance at λUV max (304 & 308) for the two strains using NR-enhanced medium, respectively

In total 60 colonies with different morphotypes (shapes/color) were selected through isolation program applied in this study using LB medium adjusted at pH 7 and incubated at 37 °C under shaking (200 rpm). All isolates were investigated for extracellular synthesis of CuNPs by incubating the cell free supernatant with CuSO4 (100mM) at equal ratio, where the biosynthesis of CuNPs was observed visually as the color change from bale blue to dark green after incubation for 48 h at 37 °C and 120 rpm. However, no color change was observed in –Ve controls, one with copper sulphate solution plus water (W), or with copper sulphate solution plus non-inoculated culture media (M), which are kept under the same conditions. Among the tested isolates only 9 isolates were capable to change the color into dark green and assigned as a positive samples (Fig. 2). A color change depends on the excitation on surface plasmon vibration of CuNPs. So, a preliminary identification of CuNPs was carried for those 9 isolates using UV-Visible spectrophotometer at the range of 200–800 nm. The two isolates coded G9 and S4c were selected as they recorded the highest SPR absorption in infra-red region (1.43 & 1.28) at λ_300−310 nm_ and in visible region (0.64 − 0.49) at λ_550−650 nm_. Further minutiae of the absorption profiles for the selected isolates (G9 & S4c) were poised in Fig. 3. In order to obtain more profound green color a medium enhanced the NR was applied, and the absorbance was measured at λ_304_ and λ_308_ (2.67 & 2.09) for G9 and S4c strains, respectively. By measuring the NR activity in LB and NR-enhanced medium, it was found an increasing in the enzyme titre (5.24 & 10.65 U/ml) and (3.29 & 5.04 U/ml), for G9 and S4c strain, respectively.

### Characterization and identification of the two selected microbial isolates

**Fig. 4:**
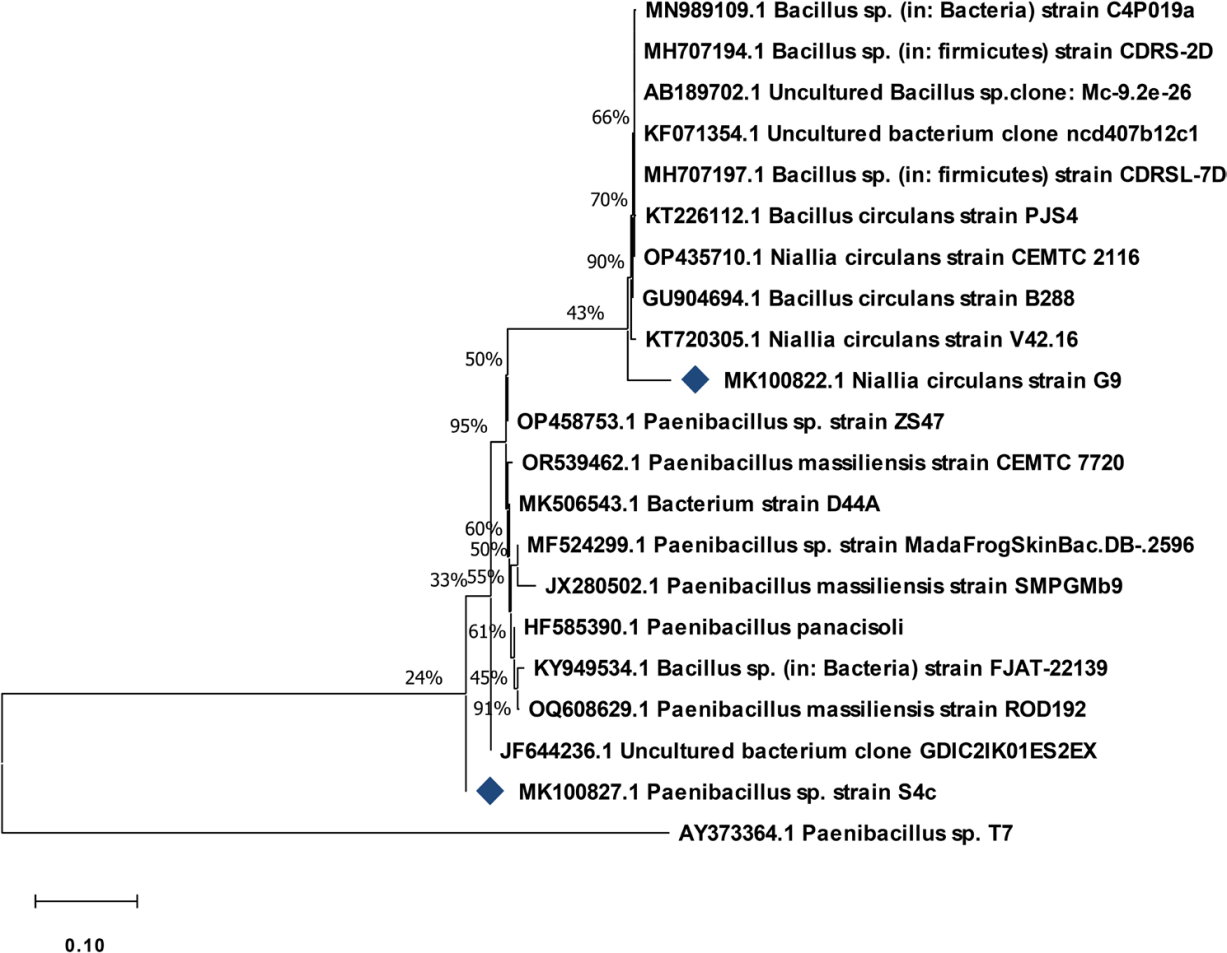
A neighbour-joining phylogenetic tree for the studied G9 and S4c strains with the nearby relatives found in GenBank

The cells of the selected isolate G9 appeared as straight, occasionally curved rods, while the cells of the S4c appeared as rod-shaped, under light microscope. The two isolates are G + ve; grow optimally at nearly neutral pH and at temperature 30, 37 ºC, respectively. According to the partial sequences of the 16s*rRNA* deposited in GenBank accession nos MK100822.1 & MK100827.1, the G9 strain nominated as *Niallia circulans* and S4c strain identified as *Paenibacillus* sp, respectively (Fig. 4).

### Factors affecting the biosynthesis of CuNPs by *Niallia circulans* G9 and *Paenibacillus* sp, S4c strains

**Fig. 5:**
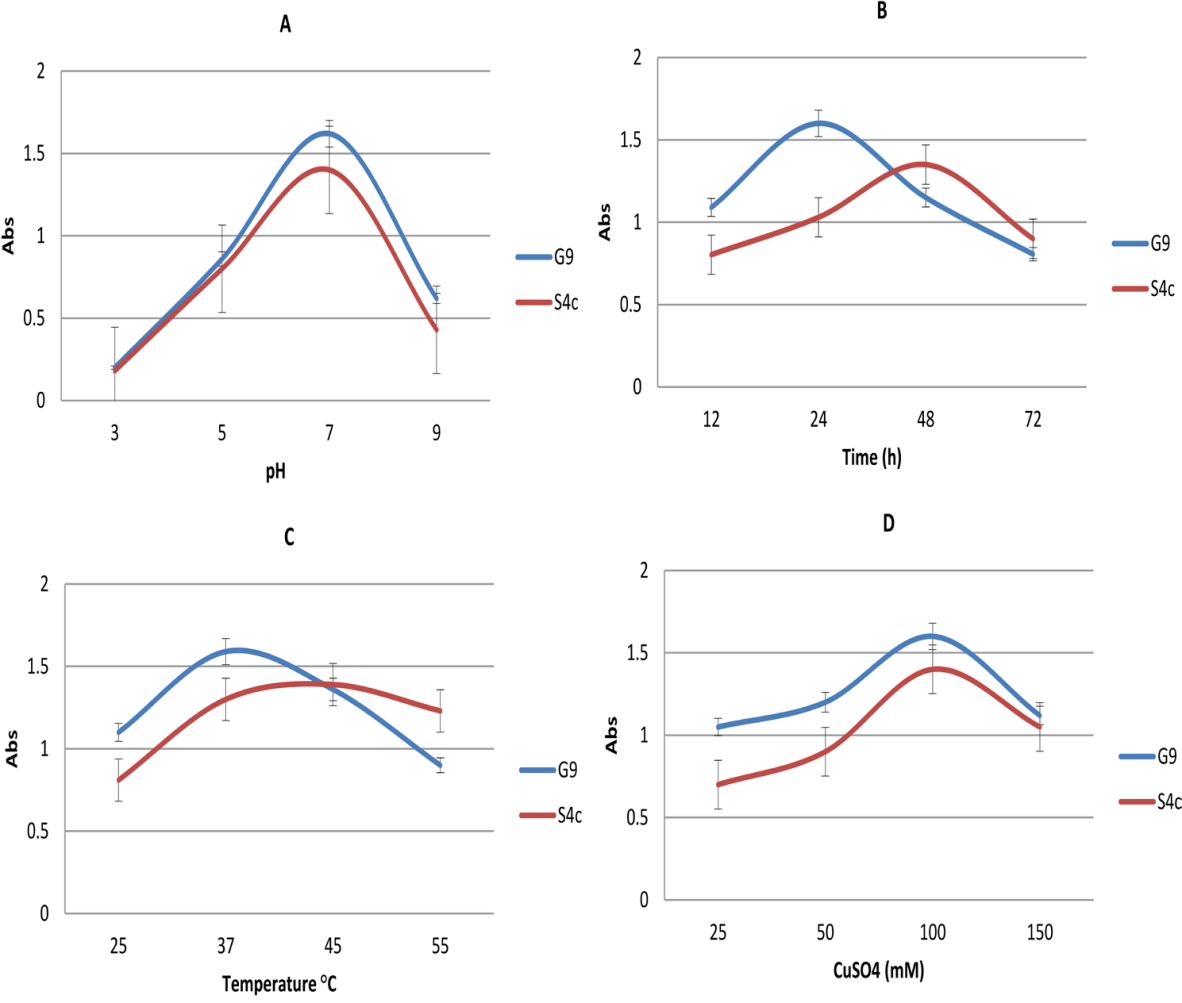
Effect of pH **(A)**, reaction time **(B)**, temperature ºC **(C)**, and metal ion concentration of CuSO4 **(D)** on the CuNPs biosynthesis by *Niallia circulans* G9 and *Paenibacillus* sp. S4c strains

The impact of pH in the CuNPs biosynthesis was evaluated under different pH values (pH 3–9), and then demonstrated in Fig. 5A. The acidic condition decreases the rate of reduction of copper ion into copper atom; this was recognized by slight change in the SPR and the color-reaction. The alkaline condition precipitates the copper and makes copper ion unavailable for reduction. However, a neutral condition showed a maximum reduction of copper ions and provides maximum synthesis of CuNPs for both tested strains.

The impact of time reaction in the CuNPs biosynthesis was demonstrated in Fig. 5B, in the case of *Bacillus circulans* strain G9, the absorbance and color of the reaction reach the maximum after 24 h, whereas in the case of *Paenibacillus* sp. strain S4c, the maximum appear after 48 h.

According to the data denoted in C section of Fig. 5C, the optimum temperatures for the production of CuNPs were at 37 °C & 45 °C by *Bacillus circulans* G9 and *Paenibacillus* sp. S4c, respectively.

To understand the influence of the copper- metal concentrations on CuNPs biosynthesis, different concentrations 25, 50, 100 and 150 mM of CuSO4 were tested, and figured (Fig. 5D). The absorption increases by increasing the copper-metal concentration, and reached the optimum level at 100 mM by the tested strains (G9 & S4c).

### Characterization of the biosynthesized CuNPs

Preliminary characterization of CuNPs was carried out by UV-Visible spectrophotometer at range of 200–800 nm., the absorbance value of CuNPs solution after using NR-based medium was 2.7 at 304 nm and 1.36 at 600 nm for *Bacillus circulans* G9, while for *Paenibacillus* sp. Sc was 2.01 at 308 nm and 0.932 at 570 nm.

**Fig. 6:**
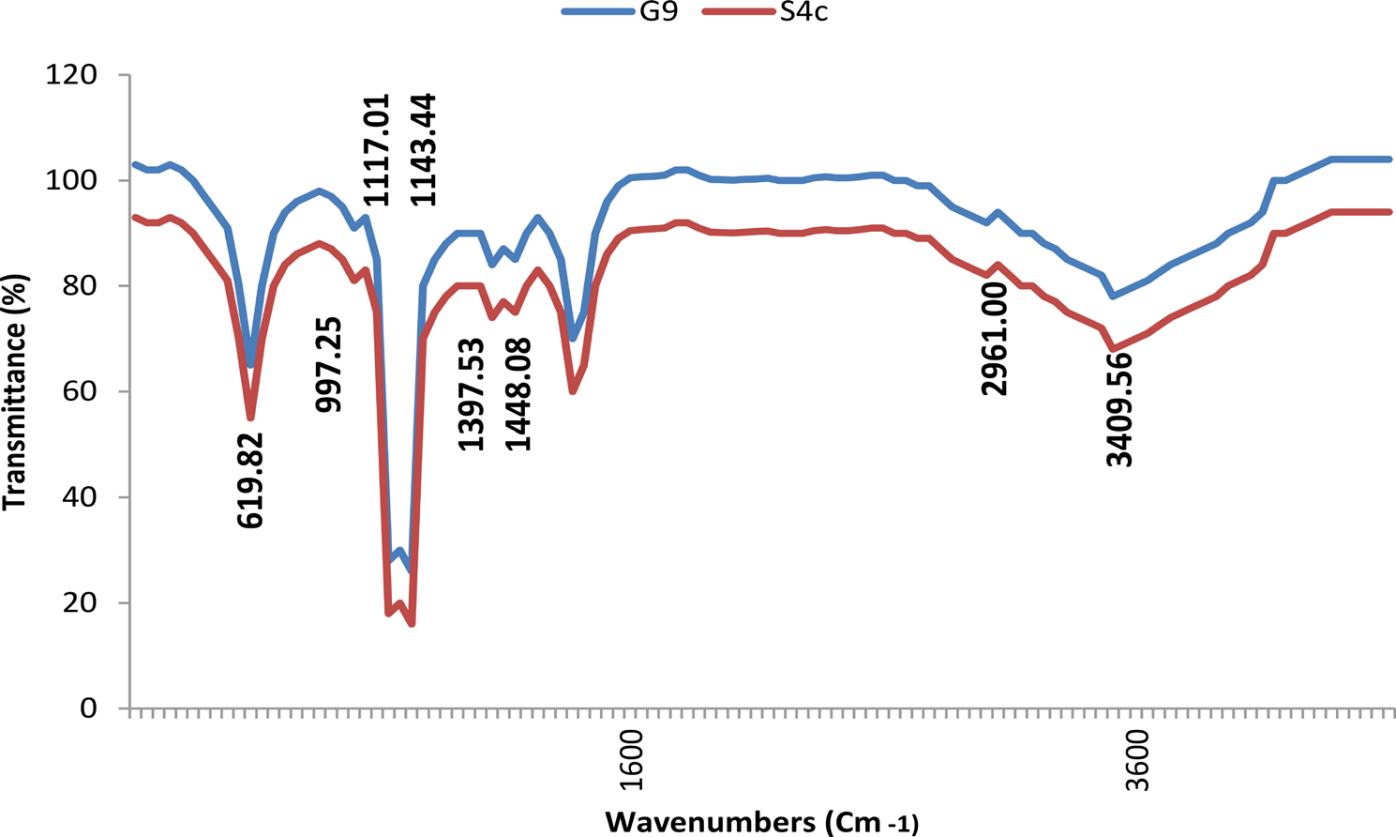
FTIR spectra recorded of the synthesized CuNPs by *Niallia circulans* G9 and *Paenibacillus* sp. S4c strains

FT-IR spectrums (Fig. 6) of the synthesised CuNPs by studied strain G9 and S4c are nealy identical. The spectrrums show band at 3409.56 cm^− 1^ (assigned to NH2 stretching vibration of primary amines and stretching vibration mode of O-H), band at 3175.95 cm^− 1^ (corresponded to O-H stretching vibration mode of carboxylic acids), band at 2961 cm^− 1^ (corresponing to aliphatic C-H stretching vibration of flavonoids/phenolic groups). The presence of bands at 2100.88 cm^− 1^ is attributed to C ≡ C stretching vibration. Whereas the band at 1638.44 cm^− 1^ (assigned to N‒H deformation found in primary amine and C = O stretching vibration in amide ≡). The FTIR band at 1448 cm^− 1^ (corresponded to C-H bending vibration of methyl group), while band at 1406.2 cm^− 1^ (corresponded to O-H bending vibration of carboxylic acid). In addition, the presence of band at 1143.43 cm^− 1^ and 117.97 cm^− 1^ (corresponds to C-N stretching of aliphatic amine). The bands seen at 997.24 cm^− 1^ (is typical to the = C-H bending due to the alkene group). Finally, the peak at 619.82 cm^− 1^ reveals the alkyne C–H bond. Also, this peak at 619.82 cm^− 1^ is related to binding Cu-NPs with oxygen from hydroxyl groups and formation of CuO.

**Fig. 7:**
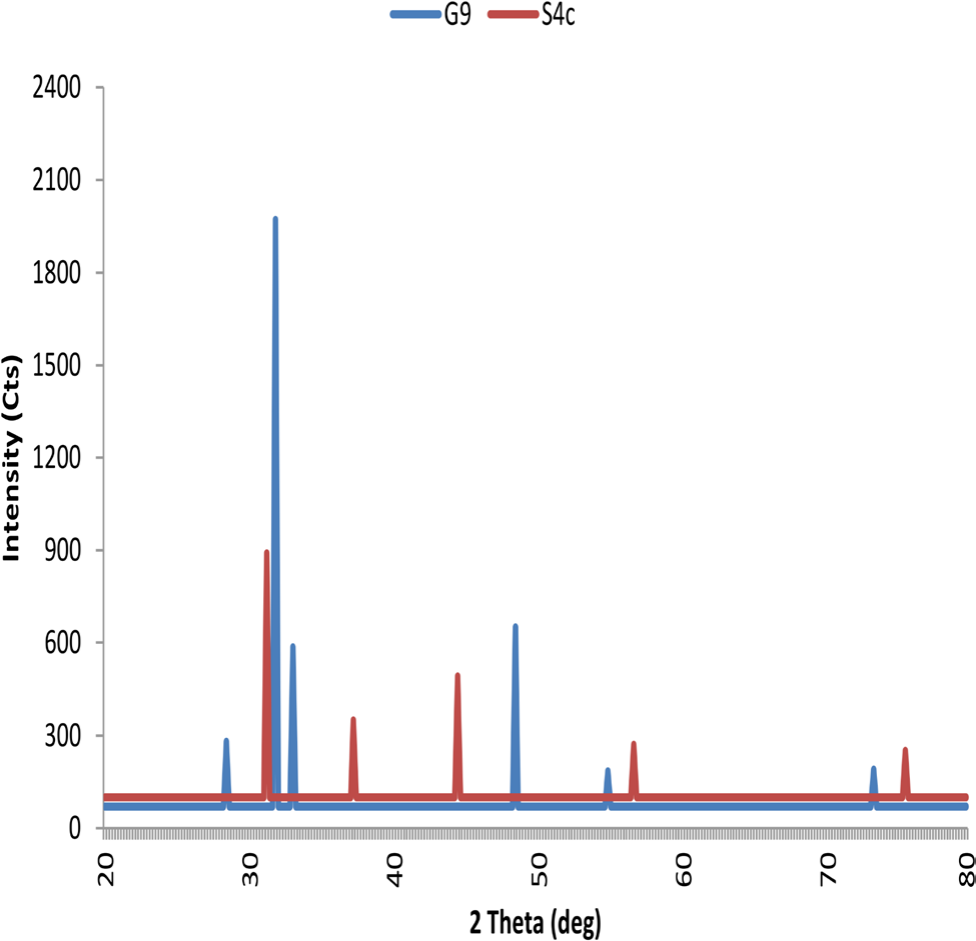
XRD pattern of CuNPs synthesized by culture free supernatant of *Niallia circulans* G9 and *Paenibacillus* sp. S4c strains

XRD examination for CuNPs from G9 strain shows distinguished peaks with 2θ value of {28.48°, 31.77°, 33.10°, 48.37°, 54.89° and 73.24°}, these peaks are assigned to the (210), (200), (220), (110), (222) and (331) reflection planes of face centered cubic structure of copper, respectively (Fig. 7). However, XRD for S4c shows distinguished peaks with 2θ value of {31.17°, 37.26°, 44.69°, 56.59° and 75.47°}, these peaks are assigned to the (200), (210), (220), (222) and (420) reflection planes of face centered cubic structure of copper, respectively (Fig. 7). The peaks appear at around 28.48–31.17° incdcate the formation of CuONPs.

**Fig. 8:**
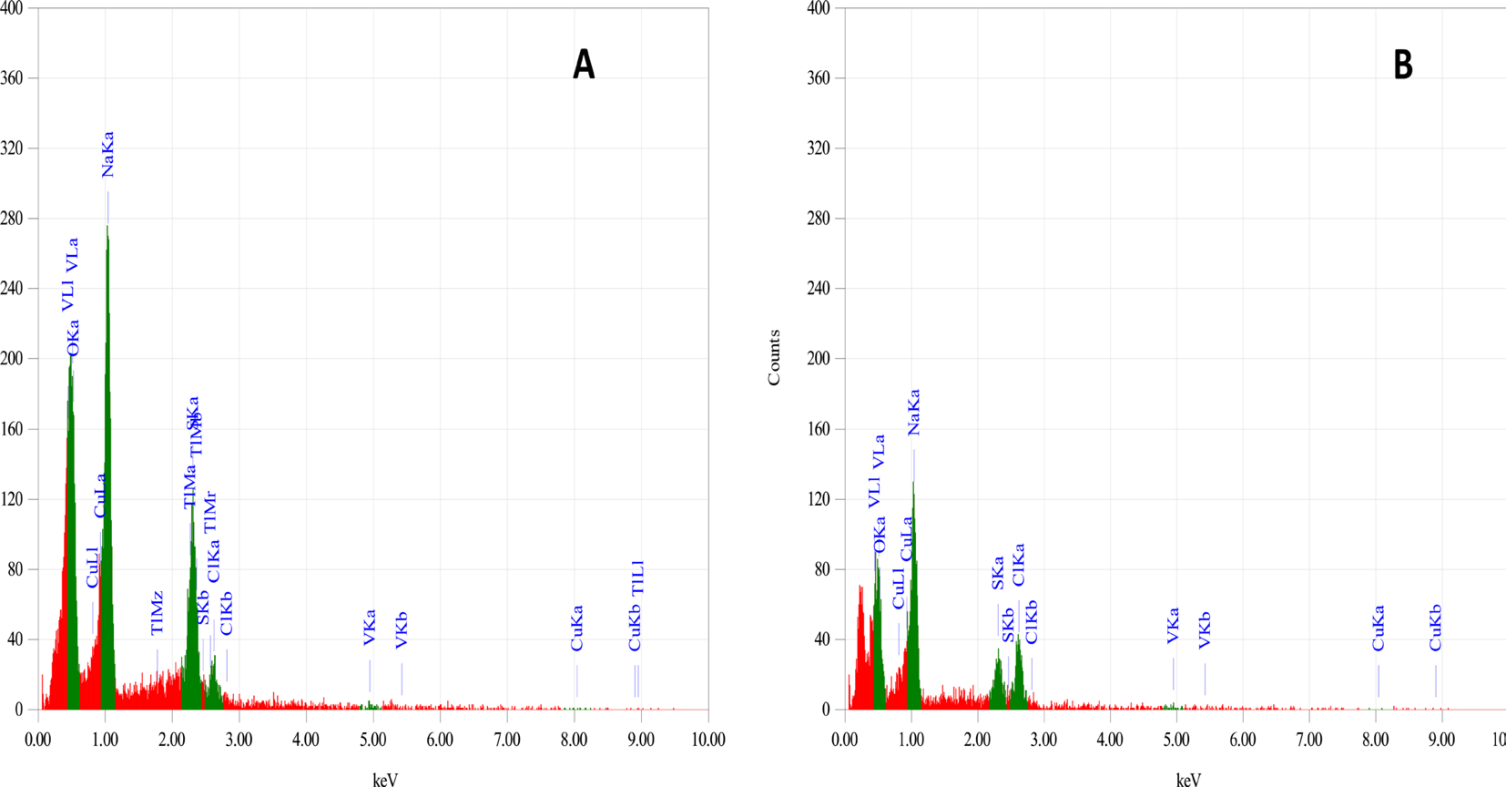
EDX spectrum of CuNPs form by *Niallia circulans* strain G9 **(A)** and *Paenibacillus* sp. strain S4c **(B)**

EDX spectroscopy analysis confirmed the presence of elemental copper by the signals in both tested CuNps samples of G9 and S4C (Fig. 8A, B). The optical absorption band peak for the produced nanoparticles was approximately at ˂1 keV, which is typical for absorption of copper. The mass of Cu in the sample was 10% and 4% for G9 and S4c strains, respectively. EDX inspections reveal the presence of peaks for O, Na, S, Cl and V.

**Fig. 9:**
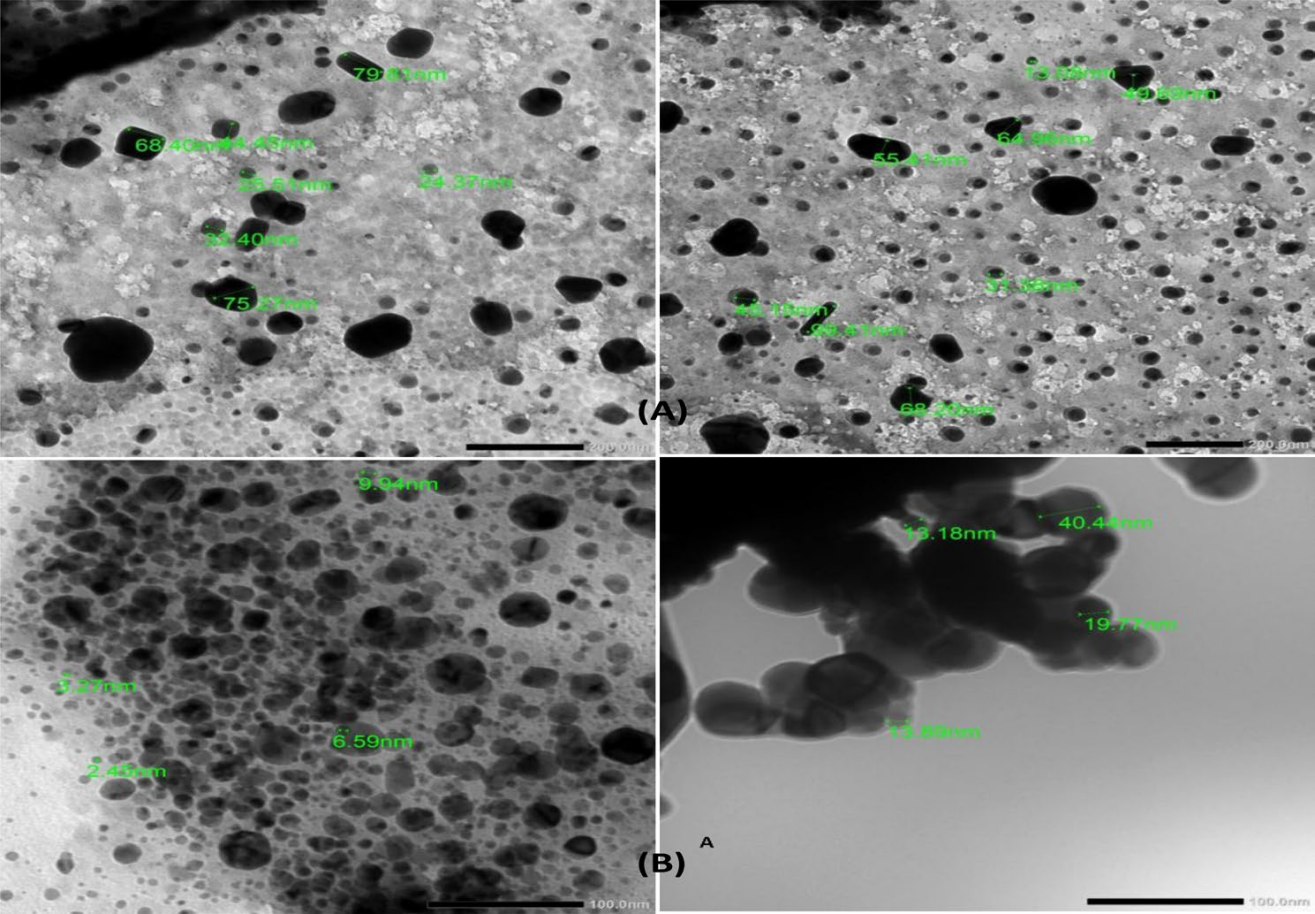
TEM analysis of the biosynthesized CuNPs produced by *Niallia circulans* strain G9 at scale a 200 nm magnification **(A)** and *Paenibacillus* sp. strain S4c at scale a100nm magnification **(B)**

TEM examination of the solution containing CuNPs (Fig. 9A, B); demonstrated the formation of spherical/hexagonal nanoparticles with a size range of 13–100 nm and spherical nanoparticles of size range 5–40 nm, for G9 and S4c, respectively.

### Antimicrobial activity of the synthesized CuNPs

In this study, the antimicrobial activity of the synthesized CuNPs against five species of human pathogenic microbes was investigated. The biosynthesized CuNPs showed significant antibacterial effects by increasing the concentrations (60–100 µg/ml).

**Fig. 10:**
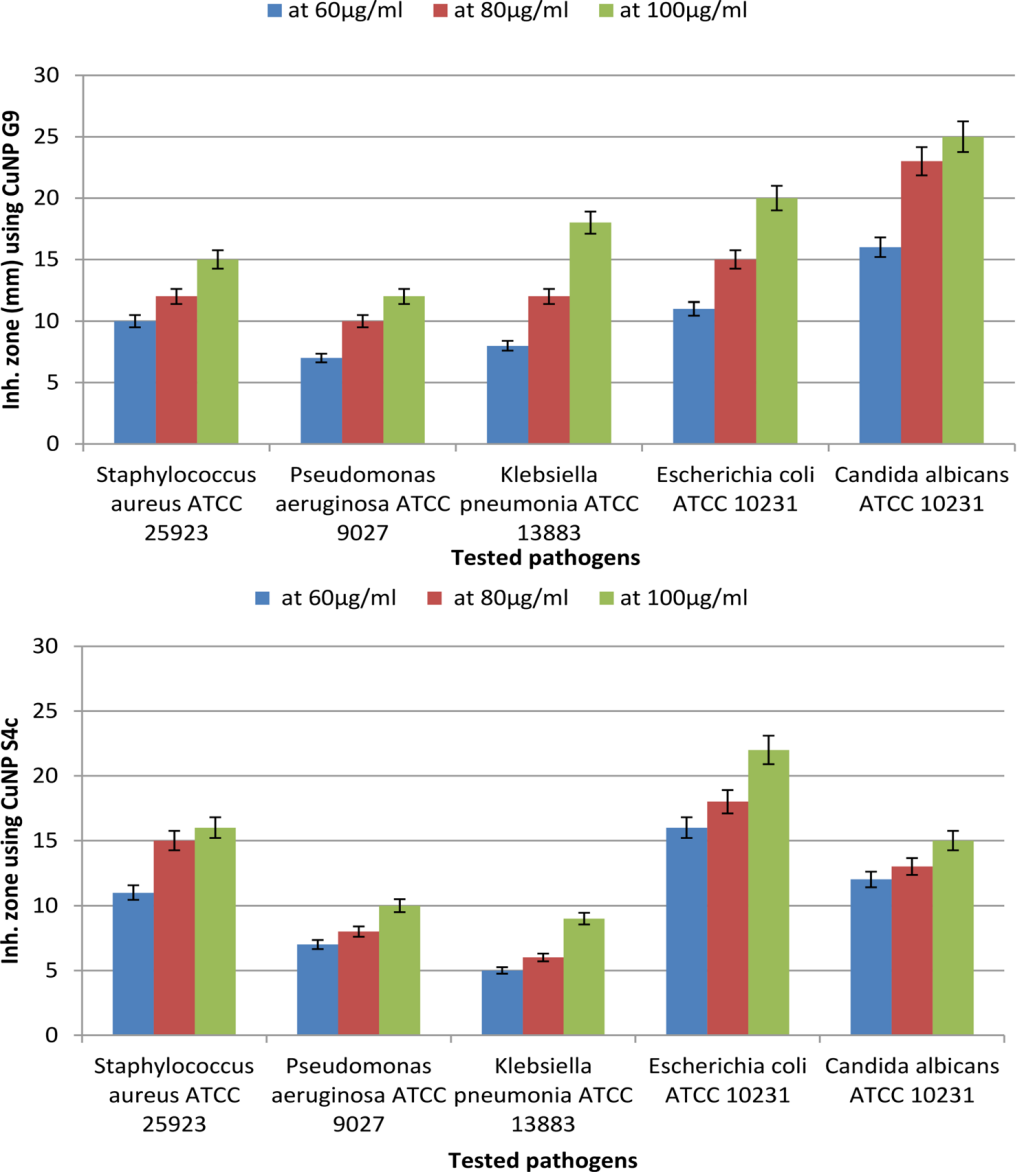
Antimicrobial activity of CuNP synthesized by *Niallia circulans* G9 and *Paenibacillus* sp. S4c strains against microbial pathogens at different concentrations

**Fig. 11:**
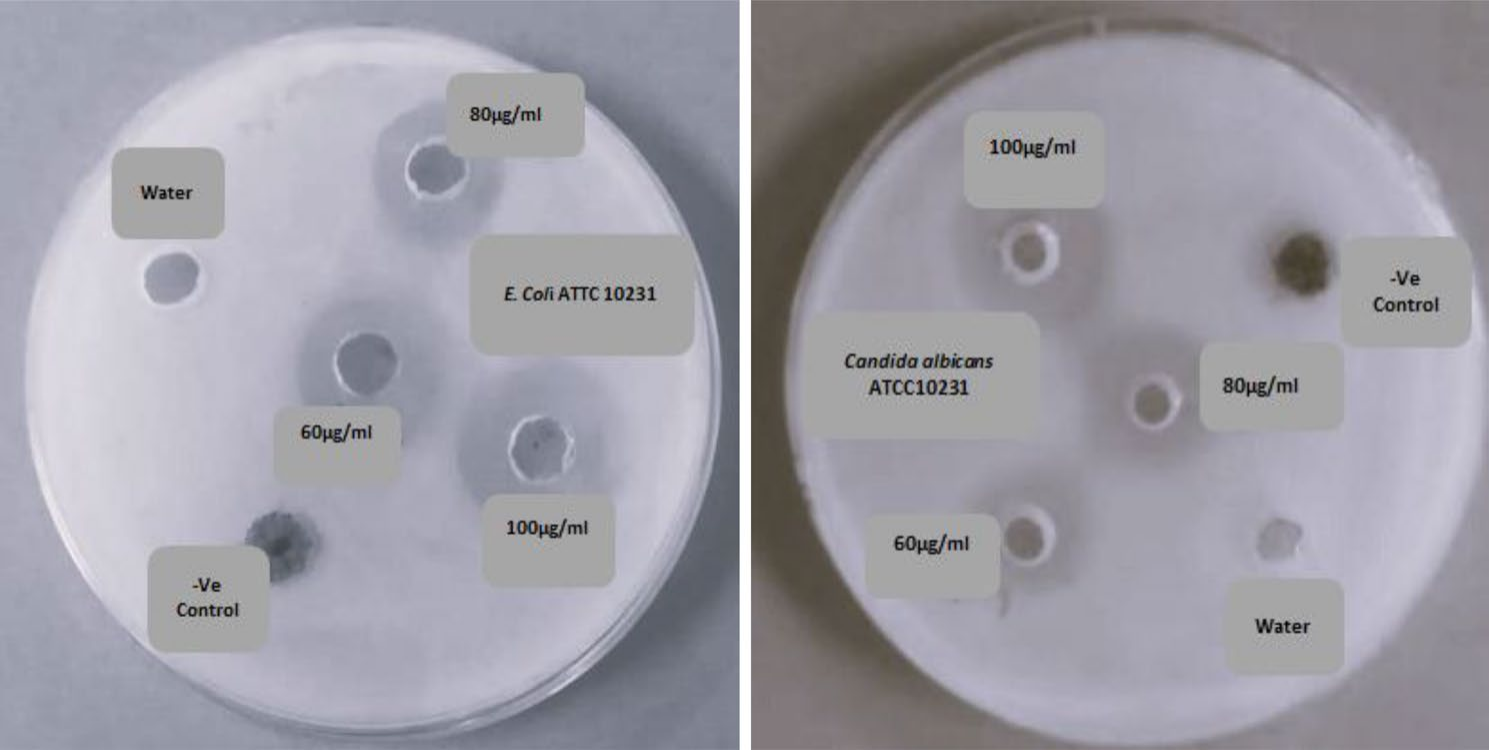
Well diffusion method of Cu NPs from S4c against *E. coli* (left) and G9 against *Candida albicans* (right) at concentrations (60, 80 & 100 µg /ml)

According to the results (Fig. 10), the yeast (*Candida albicans* ATCC 10,231) was more susceptible to the synthesized CuNPs from G9 strain than G + ve (*Staphylococcus aureus* ATCC 25,923) and G-ve (*E*. *coli* ATCC 10,231, *Pseudomonas aeruginosa* ATCC 9027 and *Klebsiella pneumonia* ATCC 13,883) bacterial strains at 100 µg/ml. On the other hand, the synthesized CuNPs by S4c strain were found more effective against *E. coli* ATCC 10,231 than the other tested pathogens at 100 µg/ml concentration. Figure 11 displays the inhibition zone containing the most potent antimicrobial activities of S4c CuNPs against *E. coli* ATCC 10,231 and G9 CuNPs against *Candida albicans* ATCC 10,231 at different concentrations (60, 80 & 100 µg/ml).

## Discussion

Extracellular production of metal nanoparticles is less expensive, simpler, and more useful than intracellular biosynthesis. Thus, culture supernatants from several isolates were collected and individually treated with copper sulphate in order to detect the color shift from bright blue to dark green (due to copper metal reduction) after 48 h at 37 °C. The change in color depends on the excitation of SPR vibrations of copper nanoparticle [36]. Preliminary identification of CuNPs was carried out by UV-Visible spectrophotometer scanning at range of 200–800 nm [28].

The typical UV-Visible absorption region for CuNPs is reported in the range from 300 to 310 nm in ultraviolet range and 570–630 nm in visual range [37, 38]. Similarly, the two isolates coded G9 and S4c recorded the highest SPR absorption at wave length 304–308 nm and 550–650 nm, respectively therefore there were selected for further investigation. These are in agreement with the work done using *Pseudomonas aeruginosa* [38] and *Enterococcus faecalis* which showed the maximum absorbance at 306 nm [39]. In this study, it was found that the magnitude of SPR absorption in UV range was more subtle than the visible range. Therefore, further experiments were carried out by measuring SPR in UV- range.

Over the years, a data base of 16 S *rRNA* gene has been constructed and it was successfully used in the differentiation of bacteria [40]. Genotypic identification emerged as a complement to establish phenotypic methods. Typically, genotypic identification of bacteria involves the use of conserved sequences within phylogenetically informative genetic targets, such as the small-subunit (16 S) *rRNA* gene [41]. In most prokaryotes, the ribosomal genes constitute an operon with the order 16–23 S–5 S and are transcribed in a single polycistronic RNA [42].

The use of molecular genetic characteristics to classify an organism and place it in a map showing the relationship between this organism and related ones, is called molecular phylogeny and a tree/map showing such a relationship is called phylogenetic tree [43]. Thus, molecular phylogeny is a combination between molecular biology and statistical techniques [44]. Accordingly the studied G9 strain designated a *Bacillus circulans* but, correctly *Niallia circulans* where it is recently transferred into genus *Niallia*. However the other selected stain (S4c) is nominated as *Paenibacillus* sp.

Previous studies have indicated that NADH and NADH-dependent enzymes are important factors in the biosynthesis of metal nanoparticles. The reduction seems to be initiated by electron transfer from the NADH by NADH-dependent reductase as electron carrier; the exact mechanism of the reduction of metal ions is yet to be elucidated for bacteria. It was documented that the bio reduction of gold ions seems to be initiated by electron transfer from NADH by NADH-dependent reductase as electron carrier. The gold ions obtained electrons are reduced to gold (Au^0^) and then to gold nanoparticles [11, 45]. This has been also excellently described in the organism *Bacillus licheniformis* which is known to secrete the cofactor NADH and NADH-dependent enzymes, especially nitrate reductase, that might be responsible for the bio reduction of Ag^+^ to Ag^0^ and the subsequent formation of silver nanoparticles [46].

NADH dehydrogenase-2 (NDH-2) was shown to promote Cu (II) reduction to Cu (I) under aerobic conditions in *E. coli* [47]. NDH-2 is present as a cytoplasmic membrane-bound enzyme; further work is required to identify the specific reductase(s) responsible.

In this study the NR activity was detected in the culture supernatant of *Niallia circulans* strain G9 (10.62 U/ml) and *Paenibacillus* sp. strain S4c (5.04 U//ml) after culturing in NR enhanced medium. These activities are higher than *Pseudomonas aeruginosa’s* NR activity (0.015 U/ml), as reported by Tiwari et al. [38]. The observed increase in absorption following the use of G9 and S4c supernatants with assessed NR activity in the creation of CuNPs suggests that the extracellular nitrate reductase enzyme in the cell-free supernatant might be responsible for the manufacture of the nano form of copper.

The extracellular synthesis offers a great advantage over an intracellular process of synthesis from the application point of view. Since the nanoparticles formed inside the biomass would have required additional step of processing for release of the nanoparticles from the biomass by ultrasound treatment or by reaction with suitable detergents. The extracellular synthesis of nanoparticle makes it possible to harness and immobilize/deposit onto desired solid support for the use of different practical purposes. In future, it would be important to understand the biochemical and molecular mechanism of the synthesis of the nanoparticles by the cell filtrate in order to achieve better control over size and polydispersity of the nanoparticles, so according to those result further investigation was carried to optimize the production of nitrate reductase enzyme.

Upon screening the variables (pH: 3–9, T: 25–55 °C, reaction time: 12–72 h, and metal conc.: 25–150 mM) affecting in CuNPs synthesis by studied strains. Quickly, the optimum conditions for biosynthesis of CuNPs can be assigned as that given the maximum SPR and change in color [48].

The pH value of the reaction media plays a significant role during the formation of nanoparticles [49]. Studies have shown that varying the pH of the reaction media tends to produce variability in shape and size of nanoparticles synthesized, this factor induce the reactivity of culture free supernatant of the selected isolates with copper ions. In this study the two strains (G9 & S4c), showed preference to a neutral condition where, this condition favours maximum reduction of copper ions and provides maximum synthesis of CuNPs. As reported by Tiwari et al., the optimum pH value for the biosynthesis of CuNPs from the cell free culture of *Pseudomonas aeruginosa* was 7.0 [38], and also cell free culture of *Pseudomonas fluorescens* at pH 7, a characteristic peak observed at 653 nm confirms the CuNPs production, indicating that neutral pH is optimal for the CuNPs formation [50].

Since time is an important aspect that supports nanoparticle synthesis and stability. It was seen that in case of *Bacillus circulans* G9, absorbance and the color of the reaction increased gradually up to 24 h then it started decreasing, while in the case of *Paenibacillus* sp. S4c it started to decrease after 48 h. This denotes that CuNPs formation occurs and with an increase in time, size reduction takes place. CuNPs biosynthesized by *Enterococcus faecalis* showed that reaction time of 24 h led to formation of well-defined CuNPs [39]. However, the reaction time for the production of CuNPs by *E. coli* was 48 h [28].

While it is generally known that reaction temperature is a crucial factor in any synthesis, it has been found that temperature is also an important factor in determining the size, shape, and yield of nanoparticles biosynthesis [51, 52]. For example, synthesis of silver nanoparticles at a reaction temperature of 25 °C via *Citrus sinensis* (sweet orange) peel extract produced particles with an average size of around 35 nm. By increasing the temperature of reaction up to 60 °C, the average particle size decreased to 10 nm [53].

In this study, the optimum temperature (37 °C) for the production of CuNPs via *Bacillus circulans* G9 is similar to researchers [39, 54], who reported that the optimum temperature for the production of CuNPs by *Serratia* sp. and *Enterococcus faecalis*, respectively, were 37 °C. While, for *Paenibacillus* sp. S4c the optimum temperature was at 45 °C, this result agree with Tiwari et al. [38] who mentioned that the optimum temperature for the synthesis of CuNPs via the cell free supernatant of *Pseudomonas aeruginosa* was 45 °C.

Metal ion concentration plays an important role in the biosynthesis of nanoparticles. Reduction of metal ions does not occur at low concentration. However, at a high concentration copper ion reduces at a faster rate resulting in the formation of macroparticles which ultimately precipitates out [1, 55]. To understand the influence of concentration of the metal, different concentrations of CuSO_4_.5H_2_O (25, 50, 100 and 150 mM) were used. The absorption was increased while increasing the concentration of silver ions from 25 mM to 100mM where it reached its optimum level. Further increases in metal concentration to 150mM copper, leads to decreasing the absorption with aggregation of CuNPs. This study concludes that the optimum copper sulphate concentration 100 mM is suitable for CuNPs production. Similarly, Chavan et al. reported that the optimum metal concentration was 100 mM for the biosynthesis of CuNPs by cell free supernatant of *Enterococcus faecalis* [39].

Different techniques can be used to analyse the nano form produced particles, herein FT-IR, XRD, EDX and TEM were followed. The band at 619 cm^− 1^ in FT-IR confirms the synthesis of CuONPs in both studied strains as stated by Kouhkan et al. [56]. The noticeable peak at 400–600 cm^− 1^ authorizes the presence of copper oxide in the biosynthesized nano particles. Similarly, Talebian et al. [21] found bands at 547 and 521 cm^− 1^ for Cu–O which confirmed the synthesis of CuONPs.

In addition, our results correlate with Tiwari et al. [38], who mentioned that the analysis of copper nanoparticles produced by *Pseudomonas aeruginosa* recorded the presence sharp peak of amide at 1642 cm^− 1^, a broad peak of N–H stretching at 3415 cm^− 1^ and a peak of N–H bending at 1362 cm^− 1^, and Nabila & Kannabiran [25], who reported a strong broad peak at 3885.07 cm^− 1^ corresponds to the O-H stretching of alcohols and phenols.

Through applying XRD analysis, the presences of sharp structural peaks in XRD patterns at 2θ value indicate the crystalline nature. The obtained pattern should be compared to Braggs’s reflection of metal nanocrystals as reported by Sher [57]. In this concern, the current results agreed with those of Ramyadevi et al. [58] mentioned that the XRD pattern recorded for the copper nanoparticles showed peaks which were indexed using JCPDS files (JCPDS card no.: 89-2838). The XRD peak positions were consistent with metallic copper. The sharp peaks of the XRD pattern indicate the crystalline nature. The peaks at 43.3165°, 50.4478° and 74.1237° corresponding to the Miller indices (111), (200) and (220), respectively represent face centered cubic structure of copper. The lattice constant of the unit cell is a = 3.615 Å and its volume is 4.7245 × 10–29 m^− 3^.

But, in contrast to Lv et al. [59], who reported that XRD patterns confirmed the formation of CuNPs by *Shewanella loihica* PV-4. The characteristic peaks were observed at 43.3◦, 50.5◦ and 74.2◦ (JCPDS 85-1326), corresponding to the crystal facets of (111), (200) and (220), respectively. Tiwari et al. [38], reported that XRD spectra of CuNPs synthesized by the culture supernatant of *Pseudomonas aeruginosa* distinguish four characteristic peaks at 2θ values of 31.398°, 45.155°, 56.197° and 75.062° corresponding to crystal facet (110), (111), (200) and (220) planes of copper.

In this study, in EDX spectrum, the nanoparticles displayed a peak approximately at ˂1 keV, which is due to the absorption of metallic copper nano crystallites corresponding to surface plasmon resonance [60], these results in contrast with the results reported by Tiwari et al. [38] where’s the peak was approximately 0.9 KeV and Chavan et al. [39] who reported a peak at 0.7 and 0.9 KeV. The mass of Cu in the studied sample was 10% and 4% for *Bacillus circulans* G9 and *Paenibacillus* sp. S4c, respectively.

However, the EDX analysis reveals the presence of peaks for O, Na, S, Cl and V. The appearance of other elements was may be due to media components or other biomolecules secreted by the bacteria [50].

According to the results of TEM CuNPs synthesized by *Bacillus circulans* G9 are nearly spherical/hexagonal in shape with an average size of 13–100 nm, these are smaller than CuNPs formed by *Pseudomonas aeruginosa* which was of size 50–300 nm [38] and those reported by Nabila & Kannabiran [25] which was of size 198 nm.

While, TEM analysis of CuNPs produced by *Paenibacillus* sp. S4c are spherical in shape of size range 5–40.44 nm. The shape of this CuNPs was smaller than that formed by *Enterococcus faecalis* which was spherical and size of 20–90 nm [39].

Screening for new antibiotics from natural sources is becoming increasingly important for the pharmaceutical industry as pathogenic bacteria are quickly becoming resistant to commonly used therapeutic agents [61]. In this study, the yeast *Candida albicans* was more affected by the synthesized CuNPs from *Bacillus circulans* G9 than the tested G + ve (*Staphylococcus aureus* ATCC 25,923*)* and G-ve (*E. coli* ATCC 10,231, *Pseudomonas aeruginosa* ATCC 9027and *Klebsiella pneumonia* ATCC 13,883) bacterial isolates at 100 µg/ml of CuNPs. On the contrary, CuNPs synthesized by *Paenibacillus* sp. S4c were more effective against *E. coli* ATCC 10,231 than the other tested pathogens at 100 µg/ml. Similarly CuNPs synthesized by *Enterococcus faecalis* culture [59] showed a high antimicrobial potential against many multidrug resistant pathogens including *Staphylococcus aureus* (MRSA), *Klebsiella pneumoniae* and *E. coli.*

## Conclusion

The current study concentrated on enhancing the production of CuNPs by two isolates, *Niallia circulans* G9 and *Paenibacillus* sp. S4c strains, which were subsequently characterised. The extracellular aliquots from NR- enriched media were used to boost CuNPs synthesis by the selected strains. The optimal conditions (T, pH, time and CuSO4 conc.) for CuNPs production were evaluated as well. The best reduction of metal copper was attained at neutral pH, and at 100 mM CuSO4 concentration. It was found, G9 strain needs shorter time and lower temperature (24 h & 37 °C) compared to S4c strain (72 h & 45 ºC) to synthesize CuNPs soundly. The application of analytics and chemical composition techniques proved the presence of CuONPs in the biosynthesized nano cupper produced by the investigated strains. According to TEM images, S4c CuNPs are smaller and have a more diverse shape (spherical, 5–40.44 nm size range) than G9 CuNPs (spherical/hexagonal, 13–100 nm size range). The biosynthesized CuNPs from S4c and G9 strains were found to be more effective against *E. coli* ATCC 10231and *Candida albicans* ATCC 10,231, respectively, in terms of antibacterial activity.

### Future prospect

Given the significant anti-pathogen efficacy of the nanoparticles created in this investigation, manufacturing and characterization are regarded as preliminary steps. The researchers’ long-term goal is to develop an appropriate formula or composite for usage in a range of disciplines such as biopesticide, water pollution treatment, and health care.

## Data Availability

No datasets were generated or analysed during the current study.
